# Impacts of Human Activities on the Composition and Abundance of Sulfate-Reducing and Sulfur-Oxidizing Microorganisms in Polluted River Sediments

**DOI:** 10.3389/fmicb.2019.00231

**Published:** 2019-02-12

**Authors:** Rui Wang, Shengjun Xu, Cancan Jiang, Yang Zhang, Na Bai, Guoqiang Zhuang, Zhihui Bai, Xuliang Zhuang

**Affiliations:** ^1^Key Laboratory of Environmental Biotechnology, Research Center for Eco-Environmental Sciences, Chinese Academy of Sciences, Beijing, China; ^2^College of Resources and Environment, University of Chinese Academy of Sciences, Beijing, China; ^3^School of Safety and Environmental Engineering, Capital University of Economics and Business, Beijing, China

**Keywords:** human activities, odorous black water, sulfate-reducing microorganisms, sulfur-oxidizing microorganisms, community structure, microbial interactions

## Abstract

Water system degradation has a severe impact on daily life, especially in developing countries. However, microbial changes associated with this degradation, especially changes in microbes related to sulfur (S) cycling, are poorly understood. In this study, the abundance, structure, and diversity of sulfate-reducing microorganisms (SRM) and sulfur-oxidizing microorganisms (SOM) in the sediments from the Ziya River Basin, which is polluted by various human interventions (urban and agricultural activities), were investigated. Quantitative real-time PCR showed that the S cycling-related (SCR) genes (*dsrB* and *soxB*) were significantly elevated, reaching 2.60 × 10^7^ and 1.81 × 10^8^ copies per gram of dry sediment, respectively, in the region polluted by human urban activities (RU), and the ratio of *dsrB* to *soxB* abundance was significantly elevated in the region polluted by human agricultural activities (RA) compared with those in the protected wildlife reserve (RP), indicating that the mechanisms underlying water system degradation differ between RU and RA. Based on a 16S rRNA gene analysis, human interventions had substantial effects on microbial communities, particularly for microbes involved in S cycling. Some SCR genera (i.e., *Desulfatiglans* and *Geothermobacter*) were enriched in the sediments from both RA and RU, while others (i.e., *Desulfofustis* and *Desulfonatronobacter*) were only enriched in the sediments from RA. A redundancy analysis indicated that NH_4_^+^-N and total organic carbon significantly influenced the abundance of SRM and SOM, and sulfate significantly influenced only the abundance of SRM. A network analysis showed high correlation between SCR microorganisms and other microbial groups for both RU and RA, including those involved in carbon and metal cycling. These findings indicated the different effects of different human interventions on the microbial community composition and water quality degradation.

## Introduction

Odorous black water is a crisis in lakes, rivers, or seashores, particularly during the summer (Feng et al., 2014). Hydrogen sulfide together with other reduced sulfur (S) compounds are the main sources of malodor in severely degraded waters (Holmer and Storkholm, 2001; Zhang et al., 2010; Watson and Juttner, 2017). In these conditions, water is highly hypoxic, leading to the death of fish and other aquatic organisms and threatening drinking water safety. During the mid-1990s and early 2000s, the phenomenon was reported in many locations, including Lake Kasumigaura in Japan, Lower Mystic Lake in the United States, Taihu Lake in China, and Subalpine Lake Garda in Italy (Zhang et al., 2010; Feng et al., 2014; Watson and Juttner, 2017). Water system degradation has a severe impact on the daily life of residents along the aquatic basin and provides an appropriate environment for pathogen growth (Feng et al., 2014; Li et al., 2014; Watson and Juttner, 2017). Thus, odorous black water induced by human urban or agricultural activities is a serious crisis, especially in developing countries where aquatic systems are more seriously and easily polluted (Kivaisi, 2001; Li et al., 2014). The phenomenon is closely related to high levels of organic matter, ammonium, and other oxygen-consuming pollutants (Feng et al., 2014; Watson and Juttner, 2017; Hu et al., 2018). However, few studies have examined microbial changes leading to the phenomenon, especially changes in microbes involved in S cycling.

Sulfur exists in a variety of reduced and oxidized compounds in inland aquatic systems (Jorgensen, 1982; Edwardson and Hollibaugh, 2017; Bao et al., 2018). S cycling, a key microbial metabolic process, is dominated by strictly anaerobic sulfate-reducing microorganisms (SRM) and sulfur-oxidizing microorganisms (SOM) (Varon-Lopez et al., 2014; Guo et al., 2016; Meier et al., 2017). SRM is an essential group for the reduction reaction of sulfate, while SOM are involved in the oxidation of sulfide in the sediments (Varon-Lopez et al., 2014; Bao et al., 2018). S cycling is connected to the cycling of other elements, such as carbon (e.g., oxidation of organic compounds and formation of organo-sulfur compounds), nitrogen (thiodenitrification), and metal (formation of metal sulfides) cycling (Edwardson and Hollibaugh, 2017; Tian et al., 2017; Bao et al., 2018). Globally, recent research has suggested that the remineralization of up to 29% of the organic matter deposited on the seafloor is facilitated by SRM (Bowles et al., 2014). In turn, hydrogen sulfide and other reduced sulfur compounds serve as electron donors for SOM or are abiotically oxidized (Bowles et al., 2014; Edwardson and Hollibaugh, 2017). Historically, S and arsenic (As) have been studied together owing to their co-occurrence in the minerals arsenopyrite, orpiment, and realgar (Edwardson and Hollibaugh, 2017; Uhrynowski et al., 2017). For example, some soluble arsenic-sulfur oxyanions (thioarsenic compounds) play important roles in As geochemistry, especially in alkaline waters (Fisher et al., 2008; Edwardson and Hollibaugh, 2017). Moreover, sulfide oxidation by SOM in aquatic sediments can be coupled to the reduction of Fe(III) to Fe(II) (Feng et al., 2014; Hausmann et al., 2016; Watson and Juttner, 2017). Thus, studying the abundance and diversity of SRM and SOM is important for revealing the roles of these microorganisms in the biogeochemical cycling of sulfur and other elements.

Both SRM and SOM differ in abundance and diversity among environments and are substantially influenced by environmental factors, such as temperature, organic matter, sulfides, geochemical gradients, and sampling depth (Varon-Lopez et al., 2014; Robador et al., 2016; Edwardson and Hollibaugh, 2017; Meier et al., 2017; Zhang M.J. et al., 2017; Zhang Y. et al., 2017). Generally, SRM are dominated by Deltaproteobacteria and Firmicutes (Haller et al., 2011; Mueller et al., 2015; Robador et al., 2016; Zhang Y. et al., 2017), while Gammaproteobacteria and Epsilonproteobacteria are considered dominant SOM on aquatic sediment surfaces (Lenk et al., 2012; Meier et al., 2017). However, some genera (e.g., *Desulfitobacterium* and *Desulfitibacter* in Firmicutes and *Pyrobaculum* in Archaea) of SRM have functional genes for sulfate reduction but cannot use sulfate as a terminal electron acceptor (Mueller et al., 2015; Edwardson and Hollibaugh, 2017). Unlike most SRM, SOM do not share a common sulfur metabolism pathway, but exploit various, partially redundant enzyme systems for the oxidation of a range of reduced sulfur compounds with intermediate oxidation states (Friedrich et al., 2005; Mueller et al., 2015). To explore the abundance and diversity of SRM and SOM in the environment, the *dsrB* and *soxB* genes, encoding the DsrB and SoxB subunit of Dsr and Sox enzyme systems in SRM and SOM, respectively, are widely used (Lenk et al., 2012; Varon-Lopez et al., 2014; Guo et al., 2016; Edwardson and Hollibaugh, 2017; Tian et al., 2017).

Various molecular methods have been used to characterize the community diversity and structure of SRM and SOM based on functional gene markers (Varon-Lopez et al., 2014; Angermeyer et al., 2016; Tian et al., 2017; Zhang Y. et al., 2017). However, traditional molecular methods are limited for characterizing diverse SRM and SOM communities for the following reasons: (i) incomplete databases of functional genes; (ii) low-throughput analysis and lack of complete cover the complex microbial community; and (iii) low amplification efficiency (Varon-Lopez et al., 2014; Angermeyer et al., 2016; Zhang Y. et al., 2017). High-throughput sequencing constitutes a powerful approach for achieving complete coverage of microbial communities (Zhou et al., 2011; Zhang Y. et al., 2017). The community structure and diversity of SRM and SOM have been effectively evaluated by the high-throughput sequencing of 16S rRNA (Haller et al., 2011; Hausmann et al., 2016; Robador et al., 2016; Edwardson and Hollibaugh, 2017; Meier et al., 2017; Tian et al., 2017). However, the community structure and diversity of SRM and SOM in odorous river sediments are unclear.

The Hai River Basin (HRB) in northern China is an important center of politics, economy, and culture (Li et al., 2014). There are eight sub-river basins in the HRB, of which the Ziya River Basin (ZRB) is the most odorous black one (Ding et al., 2016). Combined with a water shortage due to local anthropogenic activities, the ZRB has become one of the most polluted basins in China, with high levels of organic matter, NH_4_^+^-N, and sulfate, which will affect the groundwater, farmlands, lakes, and coastal areas (Li et al., 2014; Ding et al., 2016; Hu et al., 2018). Odorous rivers contain high levels of nutrients and frequently experience anoxic conditions in which sulfur transformation is a highly active cycling process (Weber et al., 2006; He et al., 2017; Sela-Adler et al., 2017; Hu et al., 2018). We hypothesized that shifts in S cycling-related microorganisms (SCM) communities are related to different levels of pollutants (Lenk et al., 2012; Feng et al., 2014; Varon-Lopez et al., 2014; Hausmann et al., 2016). Based on this hypothesis, river regions with distinct interventions might show distinct microbial community compositions. In this study, we analyzed the abundance, structure, and diversity of SRM and SOM in the sediments from ZRB polluted by different types of human intervention (urban and agricultural activities) using a high-throughput sequencing approach. Additionally, using a single copy of each SRM or SOM cell (Mueller et al., 2015), key marker genes (*dsrB* and *soxB*) were applied to quantify the abundance of SRM and SOM in different polluted regions. The results of this study provide a basis for how different human interventions influence the microbial community compositions.

## Materials and Methods

### Study Site and Sampling

Sediment samples were collected in April 2016 from an odorous river basin (ZRB) in Hebei province, China. The river could be divided into three regions, an upstream region belonging to a protected wildlife reserve with little nutrients pollution (designated RP), a midstream region localized near the city center and polluted by urban waste and sludge with high concentrations of organic matter and nitrogen pollutants (RU), and a downstream region polluted by runoff from farmlands with large amounts of sulfate fertilizer application (RA) (Supplementary Figure S1 and Supplementary Table S1) (Li et al., 2014; Ding et al., 2015). A total of 27 samples were collected from three regions (RP, RU and RA) and each region had three sampling areas to ensure the representativeness. Three samples were collected in each sampling areas as replicates. A sample (or replicate) was constituted with three fully mixed sediment cores and each sediment core was obtained by a cylindrical sampler of 20 cm in depth and 10 cm in diameter. Each sample was entirely homogenized and frozen at -80°C until parameter testing and DNA extraction.

### Sediment Parameters and DNA Extraction

Sediment samples were air-dried and pH was determined using a soil-to-distilled water ratio of 1:2. Sulfate was extracted with deionized water at a ratio of 1:5 and measured using an Ion Chromatography System with an AS14 column and an ECD50 conductivity detector (ICS-1000; Dionex, Sunnyvale, CA, United States) (Liu et al., 2014). Total S (TS) was determined using an Elemental Analyzer (Vario EL III; Elementar, Langenselbold, Germany). Ammonium and nitrate in the sediment samples were extracted with 2 M KCl and determined using a Continuous Flow Analyzer (AA3; SEAL Analytical, Norderstedt, Germany) (Liu et al., 2014). Total organic carbon was determined using a Total Organic Carbon Analyzer (Vario TOC; Elementar, Germany) (de Mora et al., 2004). Sediment parameters are shown in Supplementary Table S1. Total DNA from each sample was extracted using the MoBio Powersoil DNA Extraction Kits (MoBio, Carlsbad, CA, United States) according to the manufacturer’s protocol (Wright et al., 2008) and quantified using a Nanodrop 2000 (Thermo Scientific, Wilmington, DE, United States). The purified DNA was stored at -80°C until use.

### Quantification of 16S rRNA and Functional Genes

Quantitative real-time PCR (q-PCR) is a highly sensitive method for quantitative analyses of microbial abundance (Suzuki et al., 2000; Quillet et al., 2012; Tourna et al., 2014; Varon-Lopez et al., 2014; Tian et al., 2017). In this study, abundance was determined by q-PCR using a CFX Connect Real-Time System (Bio-Rad, Hercules, CA, United States) based on 16S rRNA, *dsrB*, and *soxB* (Supplementary Table S2). The primer set BACT1369F/PROK1492R was used for 16S rRNA quantification (Suzuki et al., 2000). Primer sets DSRp2060F/DSR4R (Wagner et al., 1998; Geets et al., 2006; Varon-Lopez et al., 2014) and soxB-710F/soxB-1184R (Beller et al., 2006; Tian et al., 2017) were used for *dsrB* and *soxB* amplification, respectively. Gene abundance was quantified in a 25-μl reaction mixture containing SYBR^®^ Premix Ex Taq^TM^ from Takara Biotechnology (Kusatsu, Japan).

### High-Throughput Sequencing

The V4–V5 regions of 16S rRNA were amplified and prepared for sequencing using the Illumina MiSeq platform with the primer set F515/R907 (Muyzer et al., 1995; Wagner et al., 1998). Purified amplicons were pooled in equimolar ratios and paired-end sequenced (2 × 300 bp) was performed using the Illumina MiSeq platform (Illumina, San Diego, CA, United States). The raw reads were deposited in the NCBI Sequence Read Archive (SRA) database (Accession Number: SRR6905915-SRR6905941). Raw fastq files were quality-filtered using Trimmomatic (Bolger et al., 2014) and merged using FLASH (Magoc and Salzberg, 2011) with the following criteria. (i) Reads were truncated at any site receiving an average quality score < 20 over a 50-bp sliding window. (ii) Sequences whose overlap being longer than 10 bp were merged according to their overlap with mismatch no more than 2 bp. (iii) Sequences for each sample were separated according to barcodes (exactly matching) and primers (allowing 2 nucleotide mismatching), and reads containing ambiguous bases were removed. Thereafter, operational taxonomic units (OTUs) were clustered with 97% similarity cutoff using UPARSE (version 7.1)^1^ with a novel “greedy” algorithm that performs chimera filtering and OTU clustering simultaneously. Taxon assignments for each 16S rRNA gene sequence were determined using the RDP Classifier algorithm^2^ against the Silva (SSU123) 16S rRNA database with a confidence threshold of 70% (Amato et al., 2013).

### Statistical Analysis

Bray–Curtis distance metrics were used to analyze OTUs for each sample, and a principal coordinate analysis (PCoA) was conducted according to the distance matrix. Cladograms were drawn using the Huttenhower Galaxy web application (Huttenhower Lab, Boston, MA, United States) with the LEfSe algorithm^3^. Linear discriminant analysis (LDA) effect size was used to elucidate differences in microbial taxa (Segata et al., 2011). An LDA score of ≥ 2 indicated an important contribution to the model. A redundancy analysis was applied to evaluate linkages between proteobacterial SCM abundance and environmental variables (Andreote et al., 2009; Varon-Lopez et al., 2014) using Canoco (Canoco 4.5; Biometris, Wageningen, Netherlands). All three correlation network analysis were constructed using the Maslov-Sneppen procedure (Maslov and Sneppen, 2002; Wang et al., 2017) and visualized using Gephi 0.9.2.^4^ Origin (version 9.1) was used to create the figures. One-way analysis of variance was performed using IBM SPSS Statistics (version 22) to evaluate significant differences (*P* < 0.05) among groups.

## Results

### Quantification of 16S rRNA and Functional Genes

**FIGURE 1:**
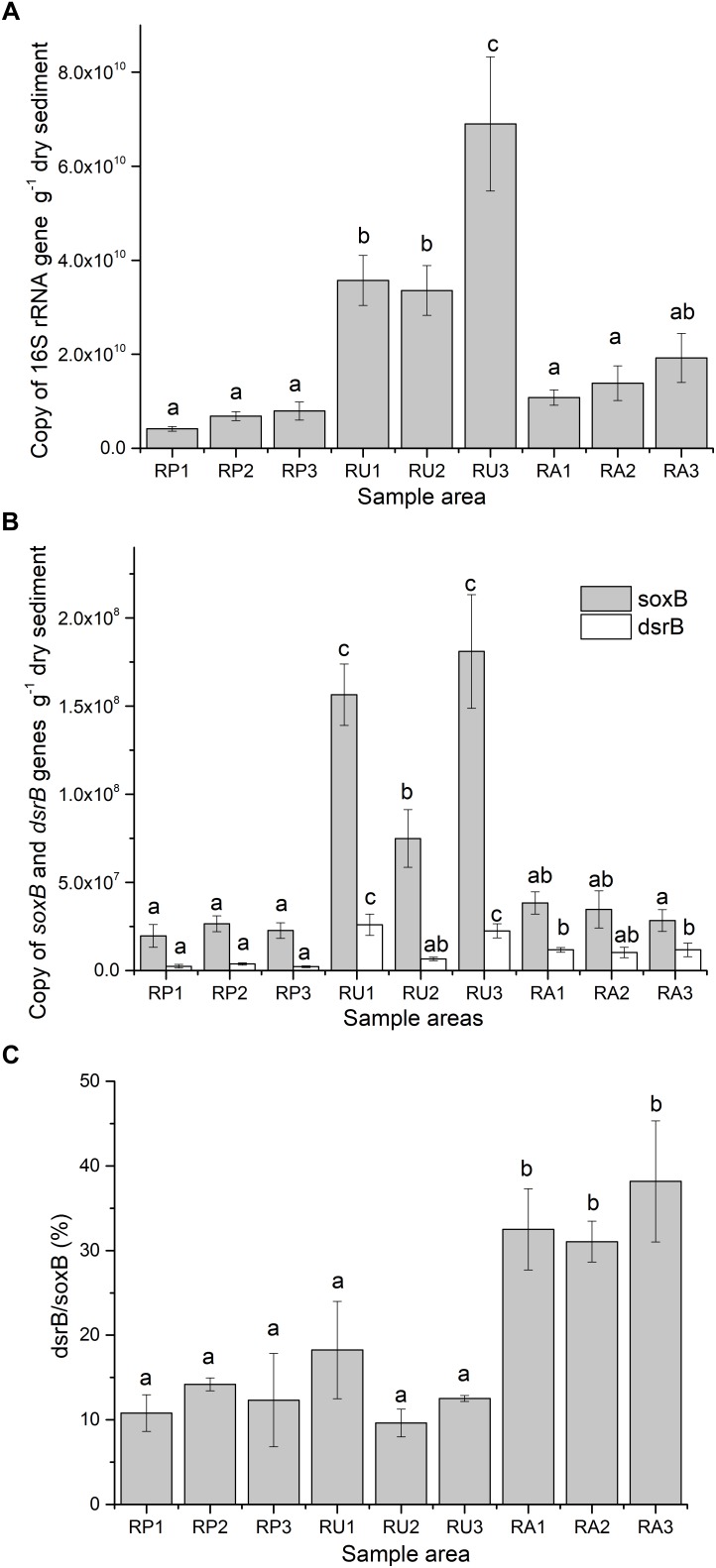
Abundance of 16S rRNA **(A)**, *aprA*, and *dsrB* gene copies **(B)** and the ratio of *dsrB* to *soxB* **(C)** in odorous river sediments with different types of human intervention. Values are represented by average values from three replications with standard errors. Different letters indicate a significant difference (*P* < 0.05). RP, protected wildlife reserve region; RU, region polluted by human urban activity; RA, region polluted by human agricultural activity.

The SRM and SOM abundances in the three regions, as determined by 16S rRNA, *dsrB*, and *soxB*, are summarized in Figure 1. Amplification efficiencies were 0.98, 0.959, and 0.95 for 16s rRNA, *dsrB*, and *soxB*, respectively (*R*^2^ = 0.999 for all genes). The 16S rRNA gene was significantly more abundant in all RU sampling areas than in all RP areas and two RA areas (*P* < 0.05). There were no significant differences between RP and RA areas (Figure 1A). In all three regions, *soxB* was more abundant than *dsrB*, ranging from 1.97 × 10^7^ to 1.81 × 10^8^ for *soxB* and from 2.21 × 10^6^ to 2.60 × 10^7^ for *dsrB* (copies per gram of dry sediment). The absolute values for both genes in RU1 and RU3 were significantly greater than those for the RP and RA areas (*P* < 0.05). Moreover, the absolute values for *dsrB* in RA1 and RA3 were significantly greater than those for the RP areas, but there were no significant differences in the absolute values for *soxB* between RP and RA (Figure 1B).

Clearer differences between the effects of human activities on SCR genes were observed when the abundance ratio of *dsrB* to *soxB* was calculated. While similar ratios were observed in the RP (12.44%) and RU areas (13.47%), significantly higher ratios (*P* < 0.05) were detected in the RA areas, with values of 32.52% for RA1, 31.07% for RA2, and 38.19% for RA3 (Figure 1C).

### Differences in Microbial Diversity With Respect to Human Interventions

Total microbial diversity was determined by 16S rRNA gene sequencing targeting the V4–V5 regions using the Illumina MiSeq platform. In total, 1020911 high-quality sequences were obtained for three regions, with an average of 37812 sequences per sample (three replicates per area, three areas per region). Random re-sampling was performed with 18,000 sequences per sample, and this resampled OTU summary table was used for further statistical analysis. An analysis of OTUs at the 97% similarity level (6954 total OTUs) showed that microbial communities were highly diverse and were dominated by Proteobacteria (20.30–39.34% of sequences) and Firmicutes (8.07–49.25% of sequences) in all areas. α-Diversity indices (Table 1) showed significant differences (*P* < 0.01) between RP and RU and between RP and RA, including indices of OTU richness (Chao index) and community diversity (Shannon index). However, there were no significant differences between RU and RA samples (*P* > 0.05). PCoA of sequence data showed significant clustering according to regions, with main principal component (PC) scores of PC1 = 22.12%, PC2 = 15.79%, and PC3 = 12.29% (Supplementary Figure S2).

**Table 1:** α-Diversity for bacterial communities in sediment samples with standard errors.

	Sobs index	Shannon index	ACE index	Chao index	Coverage
RP	1397.87 ± 282.14 A	4.89 ± 0.77 A	2487.74 ± 359.08 A	2143.92 ± 356.72 A	0.97 ± 0.01
RU	2260.36 ± 290.21 B	6.29 ± 0.48 B	3231.28 ± 369.55 B	3195.34 ± 332.78 B	0.95 ± 0.01
RA	2273.13 ± 179.49 B	6.39 ± 0.18 B	3205.62 ± 270.29 B	3194.29 ± 274.57 B	0.95 ± 0.01


*RP, protected wildlife reserve region; RU, region polluted by human urban activity; RA, region polluted by human agricultural activity. Different letters indicate significant differences (*P* < 0.05) among samples.*

Using a community bar-plot analysis, we evaluated the relative abundance of microbial communities in the three regions at the phylum level (Supplementary Figure S3). The dominant phyla of the three regions were Proteobacteria, Firmicutes, Chloroflexi, Bacteroidetes, and Actinobacteria. The proportion of Firmicutes was significantly lower in RA and RU than in RP, and the proportion of Chloroflexi was significantly higher in these regions. Both RU and RA showed significant differences (*P* < 0.05) in microbial community structure from that of RP; ten phyla were significantly different between RU and RP and between RA and RP. Significant differences between RU and RA were only obtained for four phyla, indicating a similar microbial community structure at the phylum level. Our further analyses mainly focused on the Proteobacteria in the three regions because most well-characterized SRM and SOM belonged to this phylum (Varon-Lopez et al., 2014; Angermeyer et al., 2016; Robador et al., 2016). Moreover, the Proteobacteria were more highly represented in RU and RA than in RP and we hypothesized that the difference could be related to an increase in SRM and SOM.

### Differences in Proteobacterial SCR Microbial Community Structures Among Regions

**FIGURE 2:**
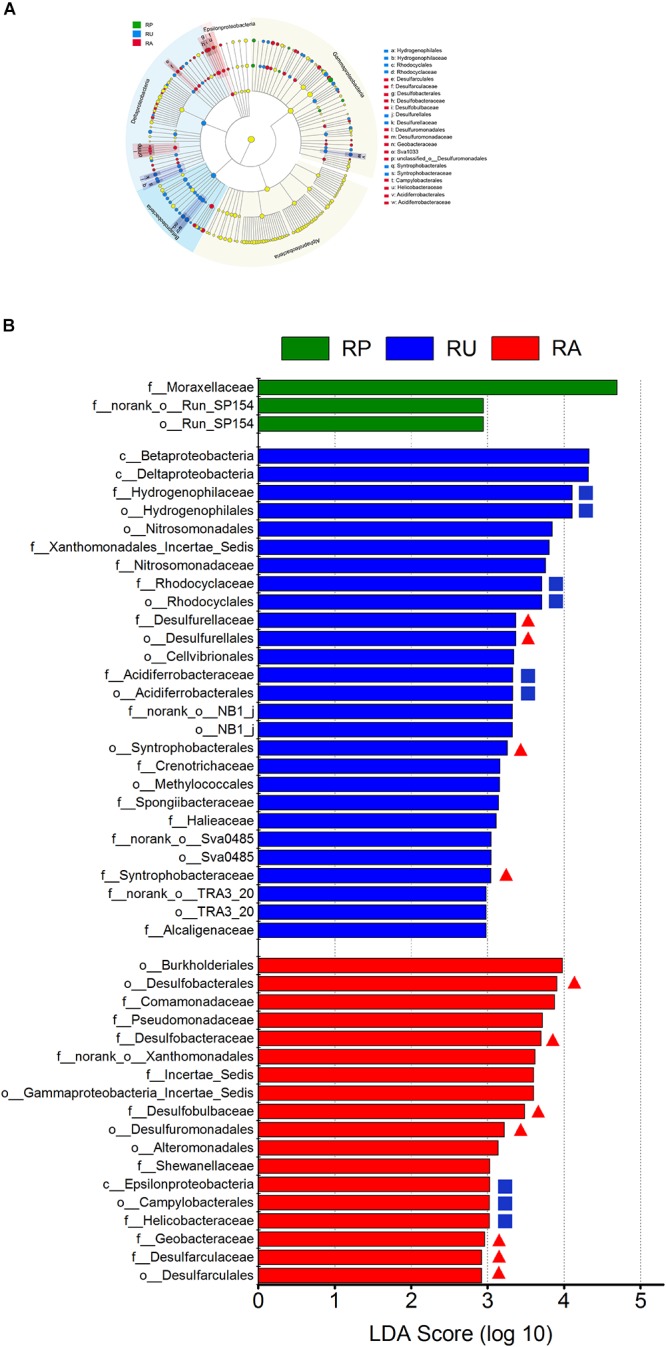
Taxonomic differences among polluted and non-polluted regions by a linear discriminant analysis (LDA) coupled with effect size (LEfSe). **(A)** Taxonomic representation of statistically and biologically consistent differences among different regions. Differences are represented by the color of the most abundant class (green: RP; blue: RU; red: RA; and yellow: insignificant). The diameter of each circle is proportional to the taxon abundance. **(B)** LDA scores were calculated by the LDA effect size, using the linear discriminant analysis to assess the effect size for each differential taxon (green: RP; blue: RU; red: RA; red triangle: sulfate-reducing microorganisms; blue square: sulfur-oxidizing microorganisms). The microbial taxa with high LDA scores (rank 50) are visualized. *n* = 9 for each region. c__: class; o__: order; f__: family.

We further investigated whether different human interventions affect community structure of SRM and SOM at various taxonomic levels (Figure 2A). Sediment microbial taxa in Proteobacteria with the greatest differences among regions are displayed in Figure 2B based on LDA scores (top 50). At the Proteobacteria level, the three regions were not distinguishable. However, RU and RA could be clearly distinguished from RP at the class level; RU was characterized by an increase in Betaproteobacteria and Deltaproteobacteria, while RA was characterized by an increase in Epsilonproteobacteria. Furthermore, clearer differences among regions were detected at the order and family levels. RU was characterized by an increase in Hydrogenophilales, Rhodocyclales, Syntrophobacterales, and Desulfurellales at the order level, while RA was characterized by an increase in Desulfobacterales, Desulfuromonadales, Campylobacterales, and Desulfarculales. At the family level, Hydrogenophilaceae, Rhodocyclaceae, Desulfurellaceae, and Acidiferrobacteraceae were enriched in RU, while Desulfobacteraceae, Desulfobulbaceae, Geobacteraceae, and Helicobacteraceae were enriched in RA. The number of characterized proteobacterial microbial phylotypes in each region was calculated (Supplementary Table S3). At the order level, 23.81% of microbes belonged to SRM in RU, and the proportion increased to 36.84% in RA. At the family level, 21.88% of the microbes belonged to SRM in RU, while the proportion increased dramatically to 83.33% in RA. Moreover, the ratio of characterized SRM to SOM was 2:5 in RU at the family level and increased dramatically to 8:2 in RA at the family level.

### Community Composition Changes and Enrichment of SCM

We further investigated differences in the relative abundances of various SCR genera among regions (Supplementary Figure S4). Diversity was greater in the SRM community structure than in the SOM community in all regions. RA showed a greater SRM community diversity than that of the other regions. In the SRM community, *Desulfobulbus* had a high relative abundance, but did not differ significantly (*P* < 0.05) among regions. H16 and Sva0081_sediment_group each had a significantly greater (*P* < 0.05) relative abundance in RU, reaching 25.21 and 15.66%, respectively, than those in the other two regions. Furthermore, as a dominant SRM genus in RP, the relative abundance of *Desulfoprunum* was 12.54%, and this was significantly greater than those in RU and RA, i.e., 3.73 and 1.80%, respectively. When considering the SOM community structure, *Thiobacillus* had a significant greater relative abundance than that of any other SOM genus in all regions, reaching 83.39% in RP, 82.69% in RU, and 76.34% in RA. Each region had characteristic SOM genera, such as *Sulfuricella* for RP, *Sulfurifustis* for RU, and *Sulfurovum* for RA. At the family level, SRM were dominated by Desulfobacteraceae and Desulfobulbaceae in all three regions. The proportion of Desulfurellaceae in the sediments from RU was significantly greater (*P* < 0.05) than those in the sediments from the other two regions. Additionally, the proportion of Desulfobacteraceae in RA was 41.03%, which was significantly greater than those in RP and RU (25.09 and 28.87%). Interestingly, Hydrogenophilaceae, a dominant SOM phylotype, maintained its overwhelming superiority over other families, i.e., greater than 75% in the sediments from all the regions. Acidiferrobacteraceae and Helicobacteraceae were the second largest phylotypes in the sediments from RU and RA regions, respectively.

**FIGURE 3:**
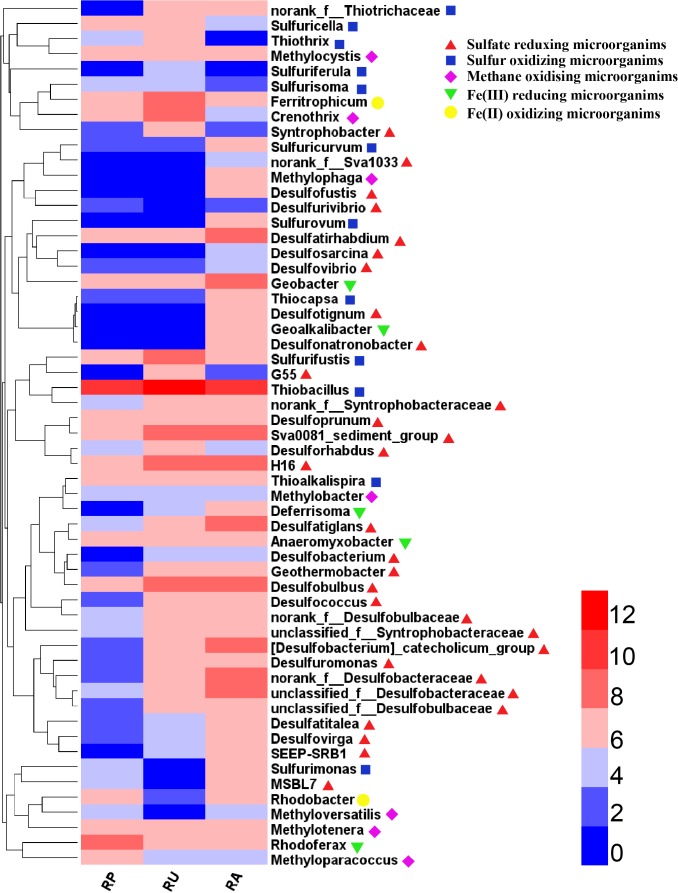
Heatmap of genera in odorous river sediments with different types of human intervention. Colored bars indicate the abundance normalized on a log2 scale, based on the color key at the top right.

To further validate differences in functional populations involved in S cycling associated with human intervention, a heat map of major genera of SRM and SOM was generated (Figure 3). Clearly, most of the SRM and SOM genera were enriched in RU and RA when compared with RP. Some SCR phylotypes, such as *Desulfatiglans*, *Geothermobacter*, and unclassified_f_Desulfobacteraceae, were enriched in the sediments from both RA and RU, while others, such as *Desulfofustis*, *Desulfonatronobacter*, and *Thiocapsa*, were only enriched in the sediments from RA. As a dominant SOM genus, *Thiobacillus* had the highest abundance in all the regions compared with other SCR genera. The environmental parameters NH_4_^+^-N, TOC, and sulfate were significantly highly correlated with the phylotype abundance of proteobacterial SRM, while only NH_4_^+^-N and TOC were significantly correlated with the phylotype abundance of proteobacterial SOM based on a redundancy analysis (Table 2).

**Table 2:** Significance of environmental variables with respect to the abundance of proteobacterial sulfate-reducing and sulfur-oxidizing microorganisms based on 16S rRNA sequences.

Variable	SRM		SOM	
	Lambda	*P*-value	Lambda	*P*-value
NH_4_^+^-N (mg/kg)	0.223	**0.001**	0.395	**0.001**
TOC (g/kg)	0.171	**0.002**	0.325	**0.007**
Sulfate (mg/kg)	0.125	**0.005**	0.116	0.074
NO_3_^-^-N (mg/kg)	0.082	0.096	0.074	0.135
TS (g/kg)	0.031	0.458	0.006	0.741


*The lambda values were obtained from forward selection in a redundancy analysis, and P-values were determined by the Monte Carlo permutation test with 999 replicates. TOC, Total organic carbon; TS, Total sulfur; SRM, Sulfate-reducing microorganisms; SOM, Sulfur-oxidizing microorganisms. Significant differences (P < 0.01) are indicated in bold.*

### Highly Complex Microbial Interactions in Polluted Regions

**FIGURE 4:**
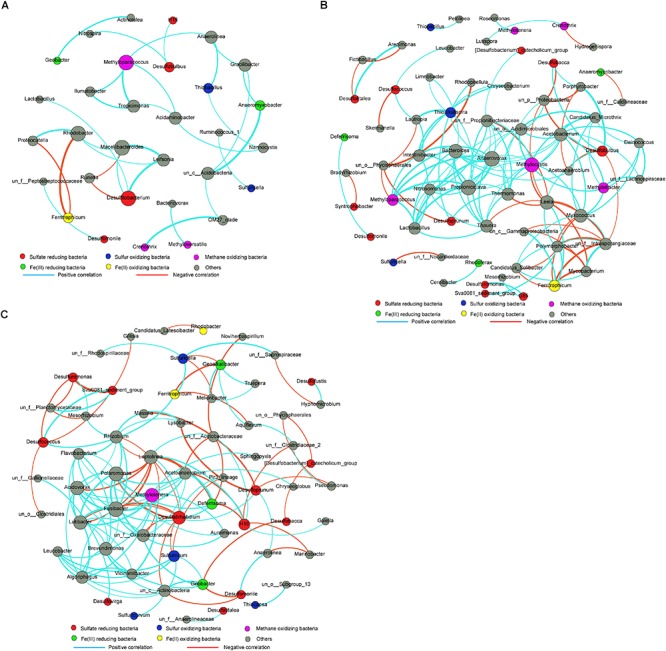
Bacterial 16S rRNA genera-based correlation network for the sediments from RP **(A)**, RU **(B)**, and RA **(C)**. A node represents a genus. A connection stands for a strong (Spearman’s *q* > 0.9 or *q* < –0.9) and significant (*P* < 0.01) correlation. Edge widths were scaled according to their weights and edge colors indicated a positive or negative correlation for the nodes they connect. The size of each node is proportional to the number of connections (the degree). *n* = 9 per group. un, unclassified.

Three correlation networks based on the relative abundance matrix of genus-level OTUs with 97% similarity were computed for the RP, RU, and RA samples. Different microbial groups involved in S, C, and Fe cycling and their interaction networks were selected for further analysis (Figure 4). Overall, the 16S rRNA gene-based networks consisted of 32 (with 31 nodes), 140 (with 65 nodes), and 139 (with 64 nodes) pairs of significant (*P* < 0.01) and robust (*q* > 0.9 or *q* < -0.9) correlations in RP, RU, and RA sediments, respectively. Modularity indices were 0.725 for RP, 0.544 for RU, and 0.496 for RA correlation networks, suggesting that all of the networks had modular structures (Liu and Conrad, 2017; Sun et al., 2018). The network structures for the three regions differed substantially in terms of composition, interaction patterns (positive, zero, or negative), and node overlap, indicating that different human interventions greatly altered the interactions among microbial phylotypes. A small and simple connected module of nodes corresponded to S cycling members in the sediments from RP, primarily including *Desulfitobacterium*, *Desulfobulbus*, and *Thiobacillus*, and these species show minimal interactions. However, in the sediments from RU and RA, large nodes directly or indirectly corresponded to members involved in S cycling, primarily including Fe(III)-reducing microorganisms (i.e., *Geobacter*, *Deferrisoma*, and *Geoalkalibacter*) and methane-oxidizing microorganisms (i.e., *Methyloparacoccus*, *Methylocystis*, and *Methylotenera*), indicating that S cycling is connected to C and metal cycling (Mueller et al., 2015; Hausmann et al., 2016; Edwardson and Hollibaugh, 2017; Liu and Conrad, 2017; Sun et al., 2018).

## Discussion

Previous studies have demonstrated relationships between SCM and environmental factors (Ramos-Padron et al., 2011; Varon-Lopez et al., 2014; Xia et al., 2014; Angermeyer et al., 2016; Pelikan et al., 2016). However, the roles of SRM and SOM in odorous river sediments resulting from various human activities are unclear. In this study, we compared the patterns of the 16S rRNA, *dsrB*, and *soxB* genes in river sediments to reveal the effects of distinct human activities on the abundance and diversity of SCM.

In the sediments of the odorous river, we found that microorganisms were significantly more abundant in all of the RU areas than in the RP and RA areas, except for RA3. The abundance of 16S rRNA was significantly (*P* < 0.001) correlated with TOC, NH_4_^+^-N, and NO_3_^-^-N (0.752, 0.827, and 0.820) in the sediment samples. Previous studies have found a similar relationship between microbial abundance and high nutriment concentrations (Varon-Lopez et al., 2014; Xia et al., 2014). The abundance of the *soxB* gene was always greater than the abundance of the *dsrB* gene in all sediment samples. Quillet observed 6.6 × 10^9^ and 4.6 × 10^8^ *dsrB* per gram of dry sediments from a salt marsh in the Medway Estuary (UK) and Tourna found 9.4 × 10^8^ *soxB* per gram of dry sediments from livestock-grazed pastures in New Zealand (Quillet et al., 2012; Tourna et al., 2014). However, in this study, the average values for *dsrB* and *soxB* were only 1.06 × 10^7^ and 6.47 × 10^7^ copies, respectively, per gram of dry sediment. This difference could be caused by variation in the depth of sediment sampled together with the availability of organic matter and the sulfate concentration (Quillet et al., 2012; Varon-Lopez et al., 2014). Various functional genes increased significantly in the areas influenced by human urban activities. Similar results were obtained in previous studies, where SRM and SOM increased significantly when nutrients were added to the sediments (Taketani et al., 2010; Xia et al., 2014; Hausmann et al., 2016). This response could be related to a decrease in oxygen and an increase in pollutants, possibly limiting the growth of aerobes (Varon-Lopez et al., 2014; Pelikan et al., 2016). However, with respect to the abundance ratio of *dsrB* to *soxB*, a significant difference was only detected in the areas influenced by human agricultural activities, while similar ratios were obtained for areas influenced by human urban activities and protected areas. A Spearman’s correlation analysis (Supplementary Table S4) indicated that the ratio was significantly correlated (*P* < 0.001) with sulfate (Spearman correlation coefficient, 0.628), indicating that sulfate was one of the main factors determining the balance between SRM and SOM communities (Varon-Lopez et al., 2014; Xia et al., 2014; Hausmann et al., 2016). These results indicated that the mechanisms underlying water system degradation differ between RU and RA.

High-throughput sequencing data showed that different human activities significantly influenced the microbial composition in odorous river sediments, with a high relative abundance of Proteobacteria and Firmicutes (Lenk et al., 2012; Varon-Lopez et al., 2014; Guo et al., 2016). As the dominant phylum, Proteobacteria always exhibits a high relative abundance across all sampling areas, accounting for 20.3–39.34% of taxa. The relative abundance of Proteobacteria was elevated in both of the polluted regions, and this could be explained by the increases in SRM and SOM, as they typically belong to Proteobacteria (Mueller et al., 2015; Meier et al., 2017; Zhang Y. et al., 2017). The increase in relative abundance in RU and RA was further quantified by a LEfSe analysis (Figure 2A). Deltaproteobacteria and Betaproteobacteria, the dominant SRM and SOM classes (Friedrich et al., 2005; Lenk et al., 2012; Mueller et al., 2015; Zhang Y. et al., 2017), were also detected in RU areas. However, not only was Alphaproteobacteria undetected in these regions, but no SCR orders or families in Alphaproteobacteria were detected in any of the regions, different from the results of previous studies (Lenk et al., 2012; Mueller et al., 2015; Hausmann et al., 2016; Robador et al., 2016; Tian et al., 2017; Zhang Y. et al., 2017). As a dominant SOM class in previous studies (Angermeyer et al., 2016; Robador et al., 2016; Zhang Y. et al., 2017), Epsilonproteobacteria was frequent in RA. Moreover, most of the SCR microbial taxa were detected in polluted regions (RU or RA), but not in RP (Figure 2B). Sulfate could stimulate the rate of sulfate reduction and the growth of SRM, especially for SRM with a relative low abundance (Hausmann et al., 2016). At the family level, we also observed eight SRM phylotypes in RA, but only two phylotypes in RU (Supplementary Table S3), which may be due to the high levels of sulfate (Strickman et al., 2016). Interestingly, microorganisms belonging to Deferribacteres were also detected in RU (data not shown). These microorganisms can reduce Fe(III) to Fe(II), which could be converted to FeS, thereby resulting in black water (Feng et al., 2014; Edwardson and Hollibaugh, 2017; Watson and Juttner, 2017). These results indicated an interaction between SCM and Fe(III)-reducing microorganisms.

SRM in all the sediment samples were dominated by Desulfobacteraceae and Desulfobulbaceae in Deltaproteobacteria (Mueller et al., 2015; Robador et al., 2016; Zhang Y. et al., 2017) (Supplementary Figure S4C). SOM mainly included members of Hydrogenophilaceae, which belongs to Epsilonproteobacteria (Lenk et al., 2012; Meier et al., 2017; Zhang Y. et al., 2017) (Supplementary Figure S4D). Meanwhile, some studies also indicated that Epsilonproteobacteria is a non-negligible phylotype of SOM in aquatic environment (Yakimov et al., 2007; Wang et al., 2012; Zhang Y. et al., 2017), which is consistent with our results. At the genus level, two groups were clearly observed; group I contained the SCR genera enriched only in the sediments from RA, and group II contained the SCR genera enriched in the sediments from both RU and RA. A high sulfate level in RA seemed to stimulate growth in many genera, especially various low abundance genera of SRM (e.g., *Desulfonatronobacter*, *Desulfofustis*, and *Desulfatirhabdium*) and SOM (e.g., *Sulfurovum* and *Thiocapsa*) as determined by a Spearman’s correlation analysis (*P* < 0.01) (Hausmann et al., 2016; Strickman et al., 2016). In fact, 21 genera of SRM were significantly correlated (*P* < 0.05) with the sulfate level, accounting for 63.64% of all SRM genera. In the SRM reducing process, sulfate is reduced to hydrogen sulfide, the dissolved metal ions are precipitated as metal sulfides, and the concentration of metal ions in solution decreases. At the same time, hydrogen ions are consumed and the pH increases (Lee et al., 2014;Zhang M.J. et al., 2017). The increased pH might lead to the excess combination of Fe^2+^ and S^2-^ to FeS that is one of main substances leading to black water (Weber et al., 2006; Feng et al., 2014).

We further explored differences in microbial interactions associated with different types of human intervention. More complex correlation networks were observed for RU and RA than for RP. Additionally, much more complex correlations between SCM (i.e., *Desulfuromonas*, *Desulfatirhabdium*, and H16 for SRM; *Sulfuricella* and *Sulfurovum* for SOM) and other microbial groups could also be observed for RU and RA, but not RP. SRM utilize propionate and straight-chain fatty acids for sulfate reduction, although many phylotypes are incomplete oxidizers (Ghattas et al., 2017; Liu and Conrad, 2017; Sun et al., 2018). *Desulfobulbus* is important for sulfate reduction in freshwater and marine sediments (Laanbroek and Pfennig, 1981; Taylor and Parkes, 1983; Laanbroek et al., 1984). In the RU region, *Desulfobulbus* had more complex correlations with other microbial groups (i.e., *Desulfobacca*, *Porphyrobacter*, and *Acetoanaerobium*) than those for RP and RA. A significant enrichment of *Desulfobulbus* was also observed in the RU areas (Figure 3). Methane-oxidizing microorganisms (i.e., *Methylocystis*, *Methyloparacoccus* and *Methylotenera*) also had complex correlations with other microbial groups for RU and RA. The correlation networks were highly complex for RU and RA for microbial phylotypes involved in S and C cycling (Asakura et al., 2014; Liu and Conrad, 2017; Sun et al., 2018). It is notable that *Geobacter*, which was significantly enriched in RA, was the most important Fe(III)-reducing genus identified at the current study site (Haller et al., 2011; Sun et al., 2018). Previous studies have indicated that *Geobacter* is highly competitive in rice paddies, using ferrihydrite as an electron acceptor, and *Anaeromyxobacter* spp. use goethite as an electron acceptor (Hori et al., 2010). In the RA region, more highly complex correlations between *Geobacter* and SCM (i.e., *Desulfomonile*, *Desulfobacca*, and *Sulfurovum*) were observed, particularly positive correlations with SOM and negative correlations with SRM. Fe(III) reduction in aquatic sediments may produce a large quantity of FeS, resulting in black water (Weber et al., 2006). Collectively, these data indicate that dynamic Fe and S cycling occurs in river sediments and may be an indicator of black water.

## Conclusion

In this study, the effects of various human interventions (urban and agricultural activity) on the abundance and diversity of SRM and SOM communities were characterized in an odorous black river system. The abundance of SCR microorganisms and the abundance ratio of *dsrB* to *soxB* were particularly high in RU and RA, respectively, indicating that RU and RA affect the odorous river system via different mechanisms. High-throughput sequencing of 16S rRNA indicated that different polluted regions were characterized by different types of SCR microorganisms. In a network analysis, the correlations between SCR microorganisms and many other microbial groups were high for RU and RA, including microbial groups involved in C and Fe cycling. Our results showed the difference of microbial community composition influenced by different human interventions. It also indicated that sulfur cycling might play a crucial role in water quality degradation and exhibited different mechanisms for water quality degradation in different polluted areas.

## Author Contributions

RW, SX, and XZ designed the research and wrote the paper. RW, CJ, YZ, and NB performed the research. RW, CJ, YZ, GZ, and ZB analyzed the data.

## Conflict of Interest Statement

The authors declare that the research was conducted in the absence of any commercial or financial relationships that could be construed as a potential conflict of interest.

## Footnotes

1 http://drive5.com/uparse/

2 http://rdp.cme.msu.edu/

3 http://huttenhower.sph.harvard.edu/galaxy

4 https://gephi.org
