# Supplementary material for: Impacts of Human Activities on the Composition and Abundance of Sulfate-Reducing and Sulfur-Oxidizing Microorganisms in Polluted River Sediments
Source: Front Microbiol. 2019 Feb 12;10:231. doi: 10.3389/fmicb.2019.00231 (PMC6379298; doi:10.3389/fmicb.2019.00231)
Supplement: Supplementary file 1 [file Data_Sheet_1.PDF]

Table S1. Characteristics of river sediment at each sampling area with standard errors.

	pH	TOC (g/kg)	NH_4^+-N (mg/kg)	NO_3^--N (mg/kg)	Sulfate (mg/kg)	TS (g/kg)
RP1	7.65±0.02 a	0.07±0.01 a	14.29±1.20 a	38.23±7.52 a	208.2±18.47 ab	0.25±0.05 a
RP2	7.6±0.01 a	0.24±0.01 a	65.13±2.08 a	173.24±16.39 ab	266.56±3.94 ab	0.46±0.03 a
RP3	7.5±0.01 a	0.24±0.04 a	60.33±6.68 a	66.65±18.14 ab	334.17±2.19 bc	0.63±0.04 ab
RU1	7.54±0.03 a	0.88±0.16 b	527.3±78.57 b	765.86±99.39 ab	157.91±6.62 a	0.54±0.04 ab
RU2	7.43±0.01 a	0.46±0.01 ab	291.02±21.12 ab	1331.49±192.86 b	300.46±11.57 b	1.76±0.15 b
RU3	7.45±0.02 a	1.11±0.10 c	419.36±23.95 b	3146.88±487.73 b	439.9±29.28 c	2.33±0.54 b
RA1	7.84±0.02 a	0.23±0.02 a	140.29±16.23 a	437.57±17.46 ab	861.07±39.46 e	0.46±0.01 a
RA2	7.76±0.01 a	0.21±0.02 a	115.9±6.43 a	205.92±53.99 ab	1156.65±97.79 f	0.29±0.03 a
RA3	7.83±0.06 a	0.16±0.02 a	79.52±9.00 a	456.05±59.50 ab	725.92±29.24 d	0.58±0.04 ab

RP: Protected wildlife reserve region; RU: Region polluted by human urban activity; RA: Region polluted by human agricultural activity; TOC: Total organic carbon; TS: Total sulfur.

Different letters indicate significant differences ($P < 0.05$) among sampling areas.
