# Supplementary material for: Impacts of Human Activities on the Composition and Abundance of Sulfate-Reducing and Sulfur-Oxidizing Microorganisms in Polluted River Sediments
Source: Front Microbiol. 2019 Feb 12;10:231. doi: 10.3389/fmicb.2019.00231 (PMC6379298; doi:10.3389/fmicb.2019.00231)
Supplement: Supplementary file 2 [file Data_Sheet_2.PDF]

Table S2. Primer sets and programs used for gene amplification.

Target gene	Primer set	Sequence	Thermal cycling profile	No. of cycles
16S rRNA	BACT1369F<br>PROK1492R	5’- CGGTGAATACGTTCYCGG -3’<br>5’- GGWTACCTTGTTACGACTT -3’	94°C/15 s, 56°C/30 s, 72°C/30 s	40
dsrB	DSRp2060F<br>DSR4R	5’- CAACATCGTYCAYACCCAGGG -3’<br>5’- GTGTAGCAGTTACCGCA -3’	94°C/15 s, 54°C/30 s, 72°C/30 s	40
soxB	soxB-710F<br>soxB-1184R	5’- ATCGGYCAGGCYTTYCCSTA -3’<br>5’- MAVGTGCCGTTGAARTTGC -3’	94°C/15 s, 54°C/30 s, 72°C/30 s	40
V4–V5 regions of 16S rRNA	F515<br>R907	5’- GTGCCAGCMGCCGCGG -3’<br>5’- CCGTCAATTCMTTTRAGTTT -3’	95°C/30 s, 55°C/30 s, 72°C/45 s	27
