# Supplementary material for: Impacts of Human Activities on the Composition and Abundance of Sulfate-Reducing and Sulfur-Oxidizing Microorganisms in Polluted River Sediments
Source: Front Microbiol. 2019 Feb 12;10:231. doi: 10.3389/fmicb.2019.00231 (PMC6379298; doi:10.3389/fmicb.2019.00231)
Supplement: Supplementary file 3 [file Data_Sheet_3.pdf]

Table S3. Summary of proteobacterial phylotypes characterized in different regions.

	Characterized Classes					Characterized Orders					Characterized Families				
	Phy	SCRP	Pro	SRP	SOP	Phy	SCRP	Pro	SRP	SOP	Phy	SCRP	Pro	SRP	SOP
RP	0	0	0%	0	0	3	0	0%	0	0	4	0	0%	0	0
RU	2	2	100%	1	1	21	5	23.81%	2	3	32	7	21.88%	2	5
RA	1	1	100%	1	0	19	7	36.84%	4	3	12	10	83.33%	8	2

RP: Protected wildlife reserve region; RU: Region polluted by human urban activity; RA: Region polluted by human agricultural activity; Phy: Phylotypes; SCRP: Sulfur cycling-related phylotypes; Pro: proportion of sulfur cycling-related phylotypes; SRP: Sulfate-reducing phylotypes; SOP: Sulfur-oxidizing phylotypes.
