# Supplementary material for: Impacts of Human Activities on the Composition and Abundance of Sulfate-Reducing and Sulfur-Oxidizing Microorganisms in Polluted River Sediments
Source: Front Microbiol. 2019 Feb 12;10:231. doi: 10.3389/fmicb.2019.00231 (PMC6379298; doi:10.3389/fmicb.2019.00231)
Supplement: Supplementary file 4 [file Data_Sheet_4.PDF]

Table S4. Spearman correlation coefficient for abundance and the ratio of functional genes in the sediment.

	pH	TOC	NH_4^+-N	NO_3^--N	Sulfate	TS
A-16S rRNA	-0.475*	0.752***	0.827***	0.820***	-0.003	0.663***
A-dsrB gene	0.056	0.466*	0.734***	0.731***	0.219	0.346
A-soxB gene	-0.472*	0.712***	0.824***	0.793***	-0.118	0.573**
dsrB/soxB	0.646***	-0.154	0.159	0.175	0.628***	-0.123

TOC: Total organic carbon; TS: Total sulfur; A-16S rRNA: Abundance of 16S rRNA gene; A-dsrB: Abundance of the *dsrB* gene; A-soxB: Abundance of the *soxB* gene; *dsrB/soxB*: Ratio of *dsrB* to *soxB* abundance.

*: Significant correlation (P < 0.05); **: Significant correlation (P < 0.01); ***: Significant correlation (P < 0.001).
