# Supplementary material for: Impacts of Human Activities on the Composition and Abundance of Sulfate-Reducing and Sulfur-Oxidizing Microorganisms in Polluted River Sediments
Source: Front Microbiol. 2019 Feb 12;10:231. doi: 10.3389/fmicb.2019.00231 (PMC6379298; doi:10.3389/fmicb.2019.00231)
Supplement: Supplementary file 5 [file Data_Sheet_5.PDF]

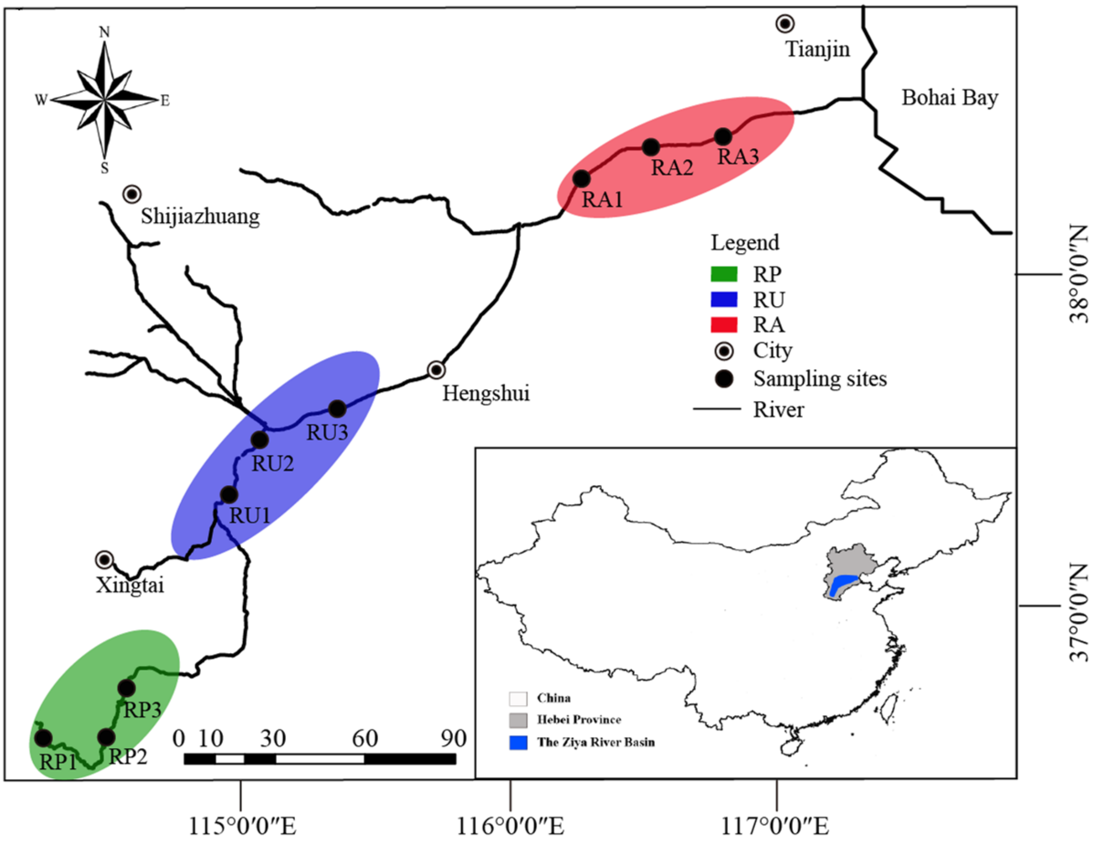

Figure S1. Sampling regions in the Ziya river basin. Latitude is indicated on the righthand side and longitude is showed on the lower edge. RP: protected wildlife reserve region; RU: region polluted by human urban activity; RA: region polluted by human agricultural activity.
