# Supplementary material for: Impacts of Human Activities on the Composition and Abundance of Sulfate-Reducing and Sulfur-Oxidizing Microorganisms in Polluted River Sediments
Source: Front Microbiol. 2019 Feb 12;10:231. doi: 10.3389/fmicb.2019.00231 (PMC6379298; doi:10.3389/fmicb.2019.00231)
Supplement: Supplementary file 6 [file Data_Sheet_6.PDF]

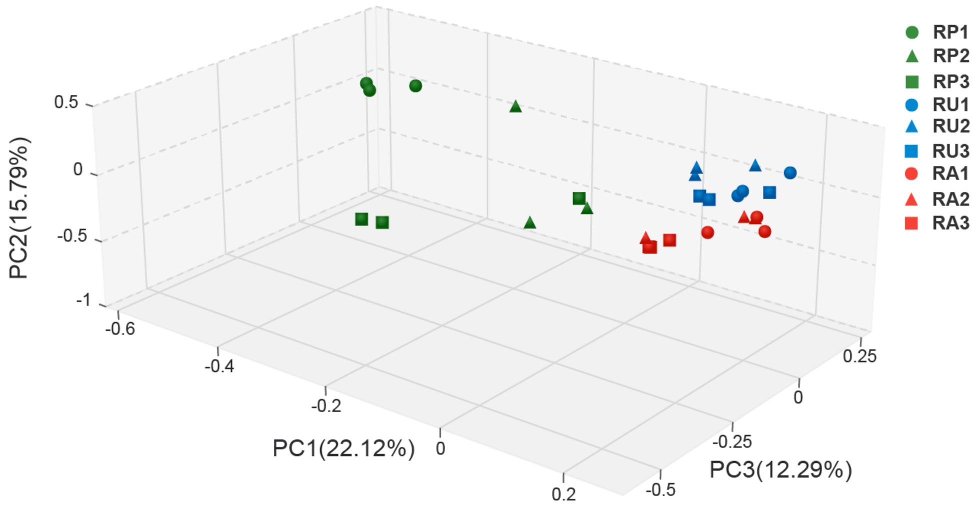

Figure S2. Principal coordinate analysis of sediment microbiota in the malodorous river. Each point represents the sediment microbiota of a replicate in RP (green), RU (blue), or RA (red). n = 9 per region.
