# Supplementary material for: Impacts of Human Activities on the Composition and Abundance of Sulfate-Reducing and Sulfur-Oxidizing Microorganisms in Polluted River Sediments
Source: Front Microbiol. 2019 Feb 12;10:231. doi: 10.3389/fmicb.2019.00231 (PMC6379298; doi:10.3389/fmicb.2019.00231)
Supplement: Supplementary file 7 [file Data_Sheet_7.PDF]

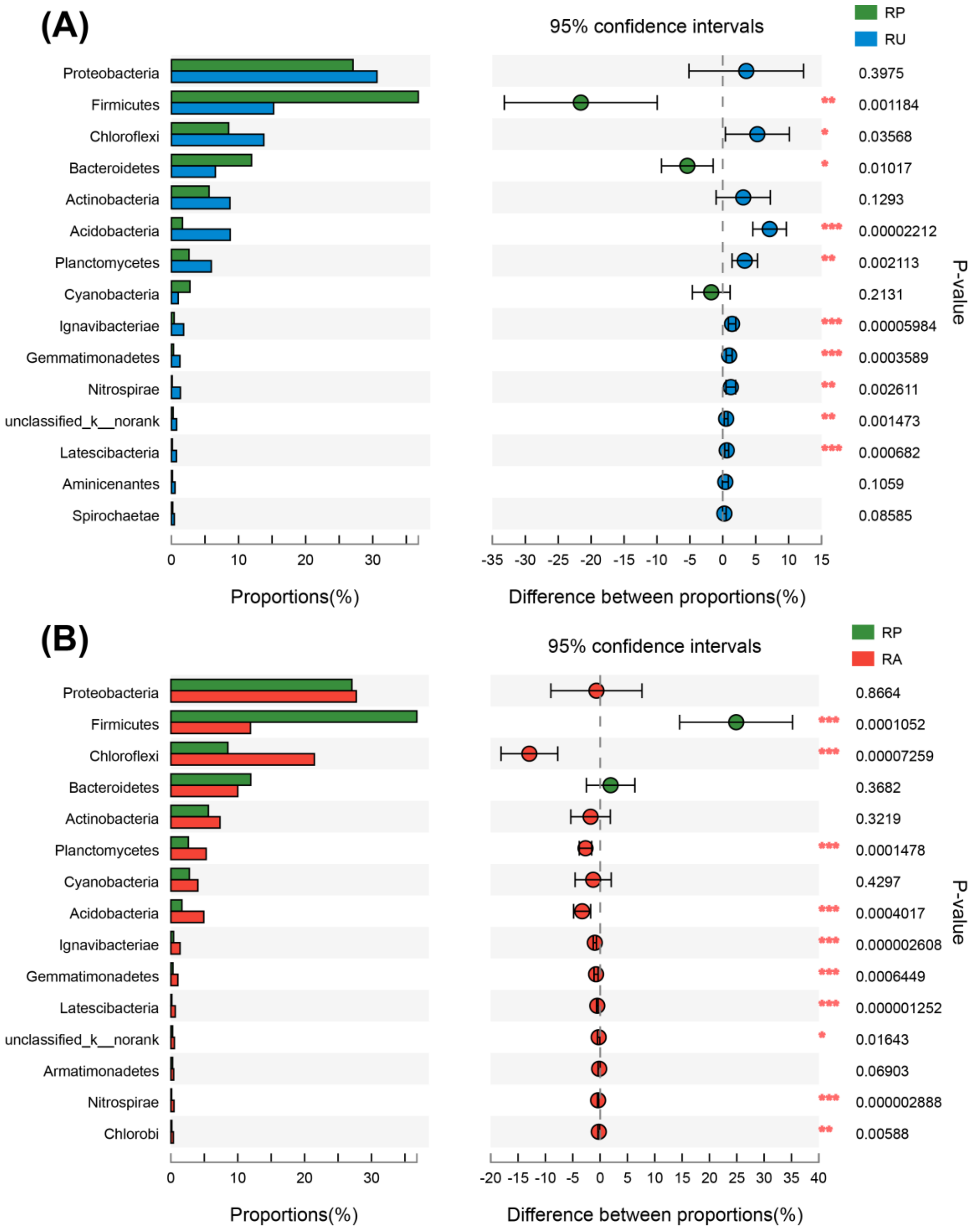
(A)
95% confidence intervals
RP
RU
Proteobacteria
Firmicutes
Chloroflexi
Bacteroidetes
Actinobacteria
Acidobacteria
Planctomycetes
Cyanobacteria
Ignavibacteriae
Gemmatimonadetes
Nitrospirae
unclassified_k__norank
Latescibacteria
Aminicenantes
Spirochaetae
0 10 20 30
Proportions(%)
-35 -30 -25 -20 -15 -10 -5 0 5 10 15
Difference between proportions(%)
P-value
0.3975
** 0.001184
* 0.03568
* 0.01017
0.1293
*** 0.00002212
** 0.002113
0.2131
*** 0.00005984
*** 0.0003589
** 0.002611
** 0.001473
*** 0.000682
0.1059
0.08585
(B)
95% confidence intervals
RP
RA
Proteobacteria
Firmicutes
Chloroflexi
Bacteroidetes
Actinobacteria
Planctomycetes
Cyanobacteria
Acidobacteria
Ignavibacteriae
Gemmatimonadetes
Latescibacteria
unclassified_k__norank
Armatimonadetes
Nitrospirae
Chlorobi
0 10 20 30
Proportions(%)
-20 -15 -10 -5 0 5 10 15 20 25 30 35 40
Difference between proportions(%)
P-value
0.8664
*** 0.0001052
*** 0.00007259
0.3682
0.3219
*** 0.0001478
0.4297
*** 0.0004017
*** 0.000002608
*** 0.0006449
*** 0.000001252
* 0.01643
0.06903
*** 0.000002888
** 0.00588

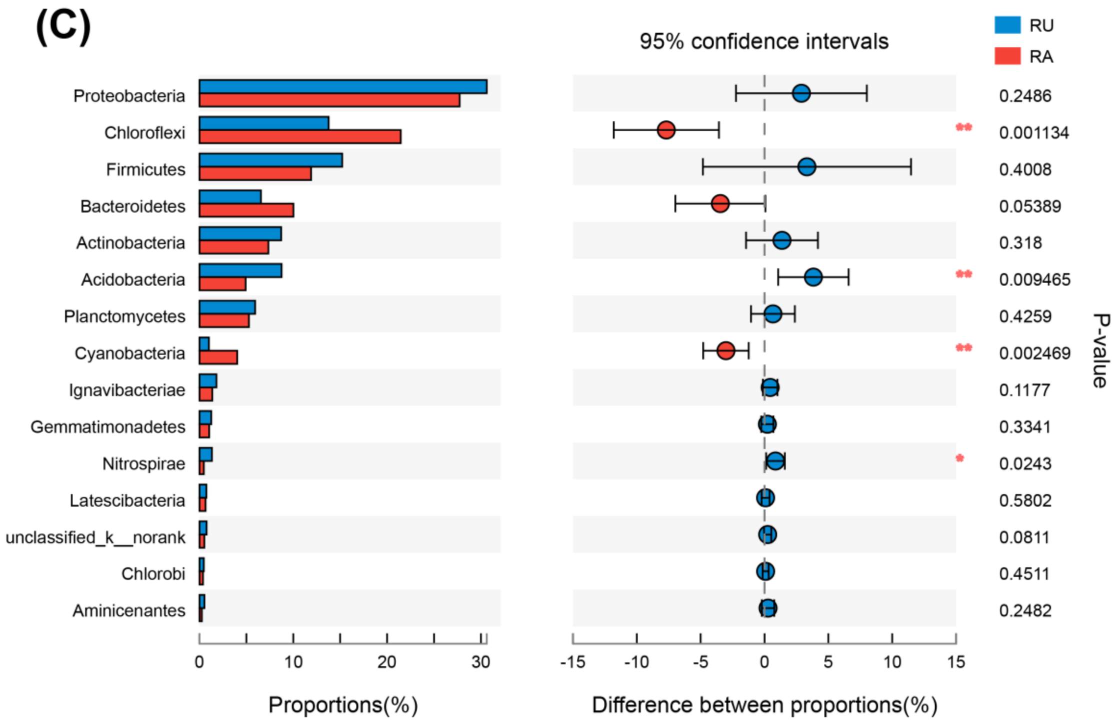

Figure S3. Phylotypes that differ significantly among sampling regions at the phylum level, RP vs. RU (A), RP vs. RA (B), RU vs. RA (C). Data are presented as the relative abundance (%) of a phylum in each region. Results were analyzed by the Mann–Whitney U test. n = 9 per region. *: P < 0.05; **P < 0.01; ***: P < 0.001.
