# Supplementary material for: Impacts of Human Activities on the Composition and Abundance of Sulfate-Reducing and Sulfur-Oxidizing Microorganisms in Polluted River Sediments
Source: Front Microbiol. 2019 Feb 12;10:231. doi: 10.3389/fmicb.2019.00231 (PMC6379298; doi:10.3389/fmicb.2019.00231)
Supplement: Supplementary file 8 [file Data_Sheet_8.PDF]

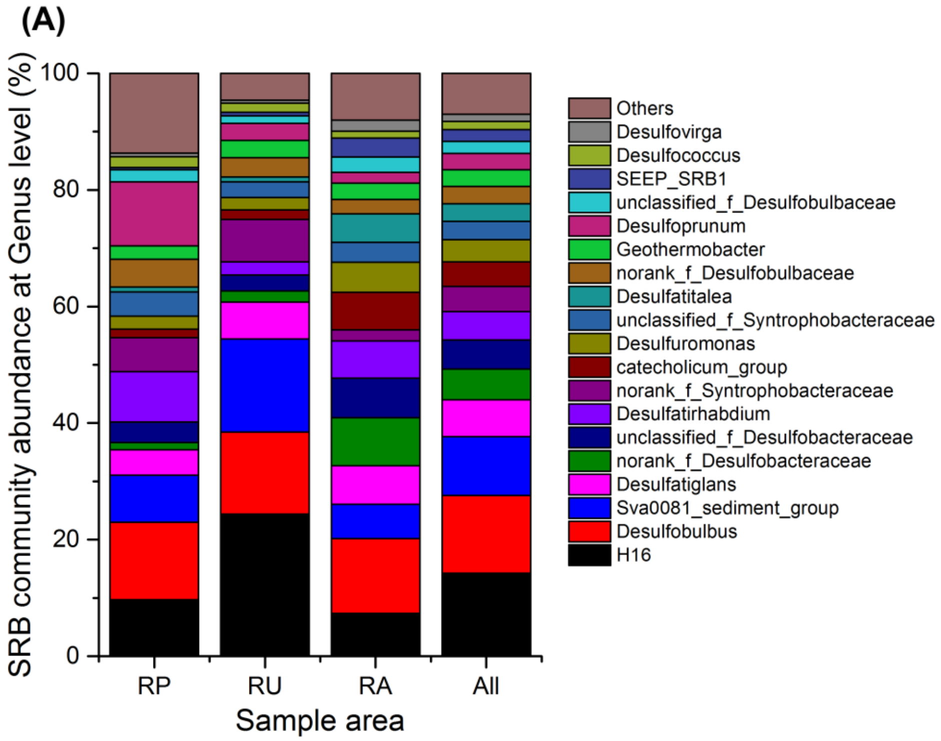

(A)
SRB community abundance at Genus level (%)
0
20
40
60
80
100
RP
RU
RA
All
Sample area
Others
Desulfovirga
Desulfococcus
SEEP_SRB1
unclassified_f_Desulfobulbaceae
Desulfoprunum
Geothermobacter
norank_f_Desulfobulbaceae
Desulfatitalea
unclassified_f_Syntrophobacteraceae
Desulfuromonas
catecholicum_group
norank_f_Syntrophobacteraceae
Desulfatirhabdium
unclassified_f_Desulfobacteraceae
norank_f_Desulfobacteraceae
Desulfatiglans
Sva0081_sediment_group
Desulfobulbus
H16

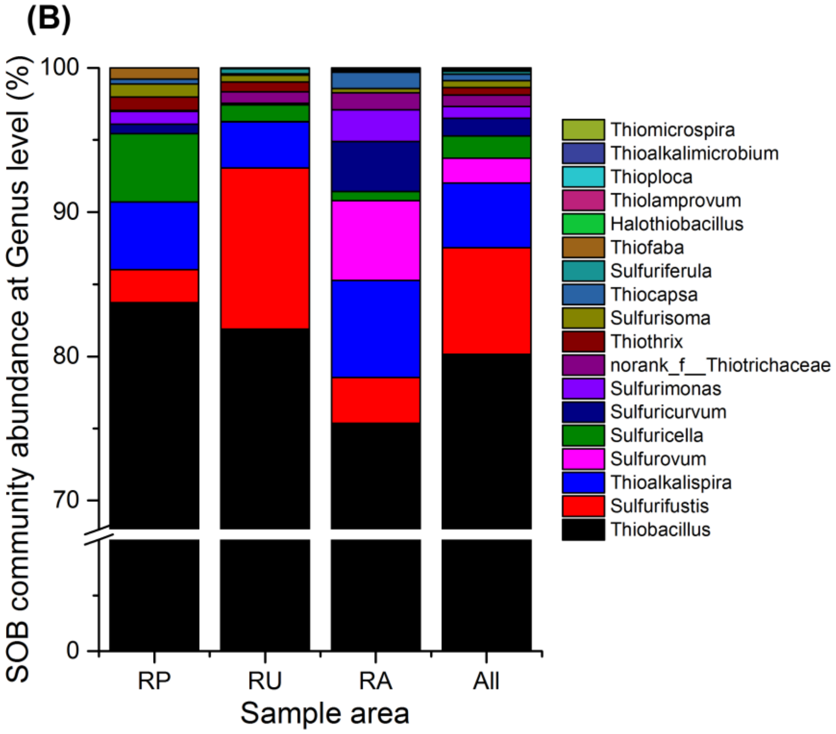

(B)
SOB community abundance at Genus level (%)
0
70
80
90
100
RP
RU
RA
All
Sample area
Thiomicrospira
Thioalkalimicrobium
Thioploca
Thiolamprovum
Halothiobacillus
Thiofaba
Sulfuriferula
Thiocapsa
Sulfurisoma
Thiothrix
norank_f__Thiotrichaceae
Sulfurimonas
Sulfuricurvum
Sulfuricella
Sulfurovum
Thioalkalispira
Sulfurifustis
Thiobacillus

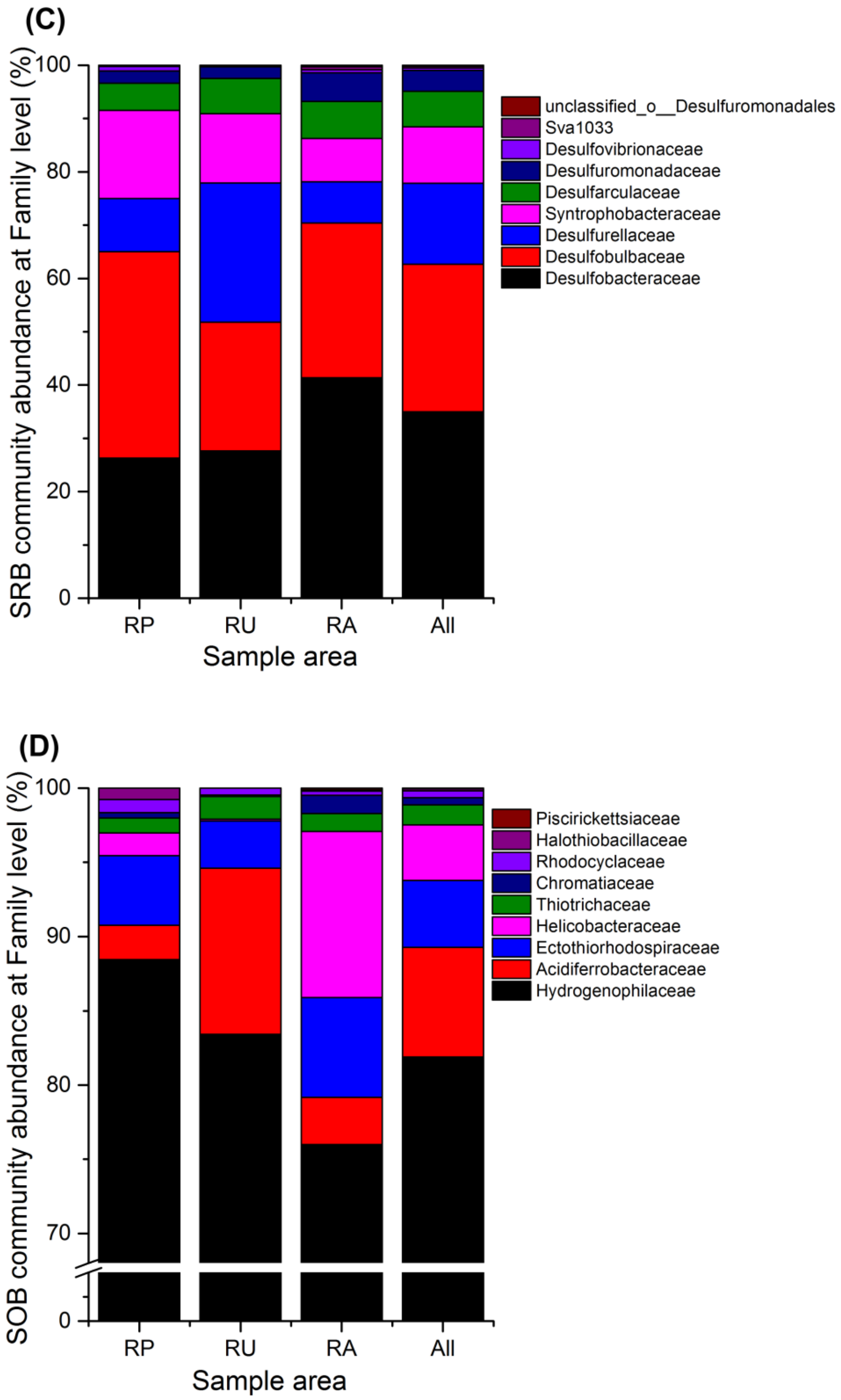

Figure S4. Community bar-plot analysis of the relative abundance of sulfate-reducing microorganisms and sulfur-oxidizing microorganisms in each sampling region at the genus (A and B) and family (C and D) levels. n = 9 for each region. SRM: Sulfate-reducing microorganisms; SOM: Sulfur-oxidizing microorganisms.
